# Education and Visual Reminders Fail to Reduce Overuse and Waste in Interhospital Transfers to a Pediatric Intensive Care Unit

**DOI:** 10.1097/pq9.0000000000000464

**Published:** 2021-08-26

**Authors:** Bernadette L. O’Neil, Jason M. Kane

**Affiliations:** From the * Section of Critical Care, Department of Pediatrics, University of Chicago, Chicago, Ill.; † Section of Critical Care, Department of Pediatrics, University of Chicago, Chicago, Ill.

## Abstract

**Introduction:**

As healthcare costs continue to rise, initiatives to reduce costs while maintaining high-quality care become a priority. Nonclinically indicated studies add to this cost, especially during interfacility transfers when studies are often repeated. Also, unnecessary evaluations add to nonmonetary costs such as pain, radiation exposure, and iatrogenic anemia. This study aimed to establish the frequency of redundant testing on interfacility transfers to the pediatric intensive care unit (PICU) and then implement an education-based quality improvement strategy for waste reduction.

**Methods:**

In the preintervention period (September 2018–February 2019), we collected data on patients transferred to the PICU from any outside facility. Investigators evaluated studies repeated within 6 hours and deemed them redundant or indicated. We then determined a rate of patients with redundant studies as the first aim. This result prompted an educational intervention focused on testing stewardship. Investigators then collected data in the postintervention period (July–December 2019) and compared the rate of redundant studies.

**Results:**

Study efforts identified 150 patients in the preintervention period and 131 in the postintervention period, establishing a 21%–25% frequency of redundant testing. Education and visual reminders failed to reduce this testing.

**Conclusion:**

This study established a baseline rate of redundant testing on transferred patients to the PICU. An educational intervention alone did not produce significant change.

## INTRODUCTION

As healthcare costs continue to rise in the United States,^1^ there is a growing imperative for local and national initiatives to decrease costs while maintaining high-quality medical care. Reducing wasteful healthcare spending is a key driver to cost containment. One well-established area of wasteful spending is excessive laboratory testing.^2^ Often patients transferred from one medical facility to another undergo repeated laboratory testing.^3–5^ In one study of emergency department patient transfers, the rate of repeated testing with previously normal results within 8 hours after arrival was 46% for CBCs and 100% for urinalysis.^4^ Furthermore, patients transferred to the inpatient setting from another facility have a higher per-patient cost than those directly admitted.^6^ Pediatric interfacility transfers from community-based hospitals to pediatric tertiary care centers are common. Thus, these patients potentially contribute to added healthcare costs relative to patients admitted via the emergency department.^7^ In addition to cost, redundant testing of laboratory and imaging studies lends to undue harm such as pediatric pain, radiation exposure, and iatrogenic anemia.^2^ Although repeat radiographic imaging or laboratory evaluations are often clinically warranted after an interfacility transfer, there may be opportunities to reduce redundant testing in patients whose recent pretransfer laboratory evaluations were nonconcerning.

To date, there have been no studies establishing the baseline frequency of repeated, unnecessary laboratory testing for pediatric interfacility transfer patients admitted to the pediatric intensive care unit (PICU). It is unclear what contribution redundant testing for newly admitted interfacility transfer PICU patients has on total hospital costs. The purpose of this endeavor to first establish a baseline rate of redundant, unnecessary laboratory evaluations for patients admitted after interfacility transfer and second to implement an educational intervention in attempts to mitigate waste related to redundant testing.

## METHODS

### Setting and Context

This report describes a prospective cohort study of interfacility transfer patients admitted to the Comer Children’s Hospital PICU at the University of Chicago. This 30-bed, quaternary care, urban, academic PICU has an annual admission volume of approximately 1,800 patients, with 30 patients per month admitted directly by interfacility transfer.

### Cohort Identification

A preintervention and postintervention convenience sample of patients younger than 19 years of age transferred directly to the PICU from any outside institution emergency department or inpatient unit comprised the patient population. Excluded patients were those with no prior laboratory evaluations performed within 6 hours before arrival, those without outside hospital records, and anyone admitted by this research team. The preintervention period was from September 2018 to February 2019. Development of the intervention plan spanned March and April 2019, with implementation in May and June 2019. Finally, the postintervention period was from July 2019 to December 2019.

### Data Collection

**Fig. 1.**
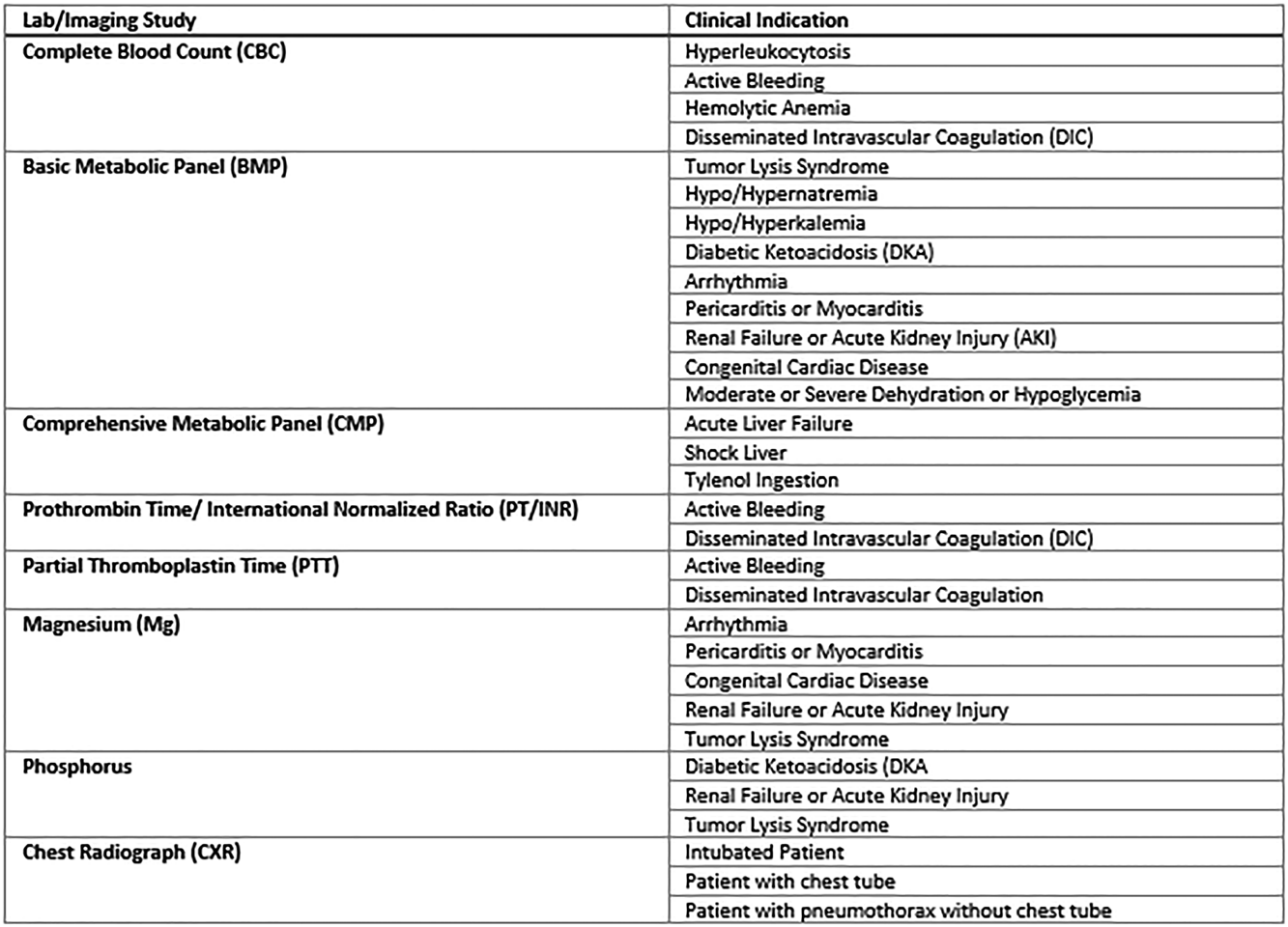
Consensus indications for clinically indicated repeat laboratory and imaging studies.

For included patients, we collected all preadmission laboratory and radiographic data relevant to the present study. The specific laboratory evaluations included complete blood count (CBC), prothrombin time/international normalized ratio (PT/INR), partial thromboplastin time, basic metabolic panel, comprehensive metabolic panel, magnesium, phosphorus, and chest radiograph (CXR). Investigators then reviewed the medical record to ascertain whether tests were repeated within six hours of the initial test result from the referring institution. To determine which scenarios would represent cases in which repeated tests were clinically indicated, the Attending Faculty in the Pediatric Critical Care Medicine section generated an a priori list of clinical indications for repeated laboratory and imaging evaluations by expert consensus opinion (Fig. 1). Investigators reviewed and attempted to apply these clinical indications for each laboratory variable repeated within six hours. If one of the clinical indications was applicable, then the repeated study was deemed “indicated.” If not, then it was deemed “redundant.” Additional demographic data, including admission major diagnostic category (MDC), were collected. We chose to assign standardized costs using the Children’s Hospital Association’s cost data coded in the Pediatric Health Information System database (Lexington, Kans.).

### Intervention

Following the initial goal of establishing a baseline rate of redundant testing, investigators implemented a testing stewardship educational intervention from May 2019 to June 2019. The intervention included a 30-minute in-person educational didactic session delivered across multiple opportunities to pediatric critical care fellows, all house staff rotating through the PICU and PICU bedside nursing staff. The principal investigator gave a three-tiered presentation with the following aims: (1) highlight the critical factors associated with excessive laboratory and imaging studies, including cost and nonmonetary harm; (2) present data on the frequency of redundant studies in the study PICU; and (3) provide instruction for repetition of clinically indicated studies. The presenter advised audience members to locate the OSH workup in one of three ways: as a paperwork packet that arrives with the patient, in the EMR if the OSH participated in our Health Information Exchange platform, Care Everywhere (Epic Systems, Inc., Verona, Wis.), or to reach out to the OSH via telephone correspondence for the data. The formal presentation was provided to the PICU fellows on May 30 at a weekly didactic session, to most of the residents on June 6 at a morning report, and to the nurses on June 26. PICU attendings were involved in creating the clinical indications for laboratory and imaging repetition; however, they were not specific targets for the didactic sessions. We felt that the residents and fellows were more directly involved in placing admission orders. The decision was made to include nurses as they were usually the initial recipient of the outside hospital paperwork and often had information regarding their patients’ workups from their hand-off communications.

Additionally, we prominently displayed a printed stewardship tool with clinical indications for repeat laboratory and radiographic studies on all PICU mobile and stationary workstations. Although a formal presentation was not repeated each month that residents rotated through the PICU, we reiterated the project goals at each rotation’s start. They were also encouraged to use the stewardship tool to enhance project adherence.

### Data Analysis

The primary outcome of interest was establishing a baseline rate of transferred patients who had redundant studies completed upon admission to a PICU and evaluating patient characteristics. A secondary outcome was the frequency of redundant testing before and after an educational intervention using a Fisher’s exact test for comparative analysis. We compared differences between preintervention and postintervention by independent sample t-test and differences in proportion by Fisher’s exact test. The median cost was compared using the Mann–Whitney U test. For the MDCs, the Pearson chi-square test was used to determine whether the distribution was similar between the 2 groups. We also created a statistical process control P-chart of the data displaying the overall percentage of transferred patients with redundant laboratory and imaging evaluations each month in the preintervention and postintervention periods. The desired direction of change was a decrease in the redundant testing. The P-chart allowed evaluation of process control stability before and after implementing the intervention and how the change affected the rate of redundant testing.

### Ethical Considerations

This project was determined to be quality improvement, and therefore, the review was waived by the Institutional Review Board at the University of Chicago.

## RESULTS

### Baseline Phase

**Fig. 2.**
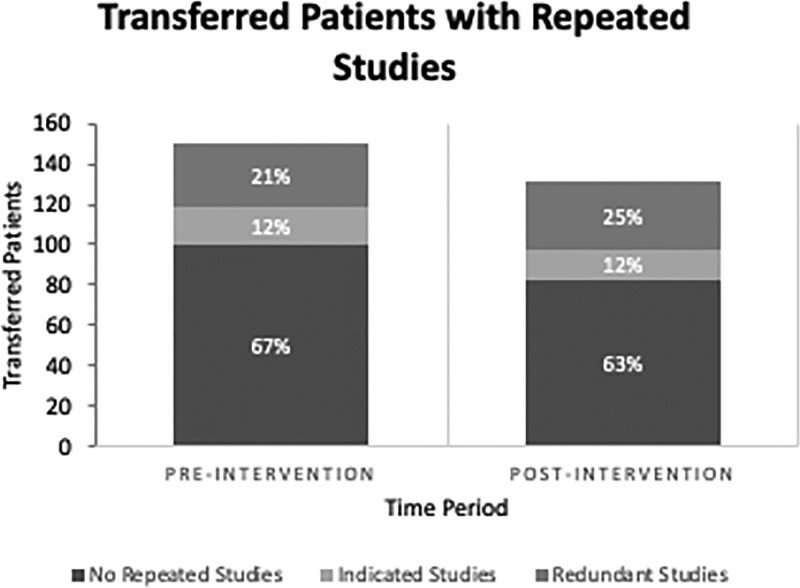
Preintervention and postintervention patient cohorts with repeated studies after transfer to the pediatric intensive care unit.

We screened 285 patients transferred to the PICU in the preintervention period, and 150 met inclusion criteria. A total of 50 patients (33%) had repeated studies, of which 31 underwent redundant testing (Fig. 2), establishing a baseline rate of redundant testing at 21%. Patients with redundant studies were significantly younger than those with indicated studies (6.87 years versus 10.98 years, *P* = 0.023). The mean cost of admission studies was also significantly different between the 2 groups, with the redundant group having a higher mean cost ($96.61 versus $62.00, *P* = 0.003). MDC distribution was also different between the 2 populations, with the most common MDC in the indicated group relating to the endocrine system (47%) and most common in the redundant group relating to the respiratory system (47.1%). There were no differences in gender, mode of transport, time of day, or admission day of the week between the groups (Table 1). Additionally, other labs and imaging characteristics for the cohort are also described in Table 2, noting that CBCs were the most common redundant study at 37%.

**Table 1.** Characteristics of Preintervention Patients with Indicated and Redundant Studies

Variable	Indicated Studies (n = 19)	Redundant Studies (n = 31)	*P*
Age (y, mean)	10.88	6.87	0.023
Sex (% female)	36.8	58	0.244
Internal transport team (%)	15.8	38.7	0.117
Daytime admission (%)	58.1	57.9	1.0
Weekend admission (%)	31.6	38.7	0.764
Cost ($, mean)	62.00	96.61	0.003
Assigned admission MDC (%)	Endocrine (47) Respiratory (21) Cardiovascular (11) Hematologic (11) Gastrointestinal (5) Neurologic (5) Infectious (0) Renal (0) Toxicology (0) Other (0)	Respiratory (42) Toxicology (16) Cardiovascular (10) Neurologic (10) Gastrointestinal (7) Endocrine (3) Hematologic (3) Infectious (3) Renal (3) Other (3)	0.023

**Table 2.** Characteristics of Repeated Studies in Preintervention Patients

Laboratory or Imaging Study	Redundant (n = 46)
CBC (%)	37
BMP (%)	13
CMP (%)	17
Mg (%)	0
Phos (%)	0
PT/INR (%)	11
PTT (%)	7
CXR (%)	15

### Postintervention Phase

**Fig. 3.**
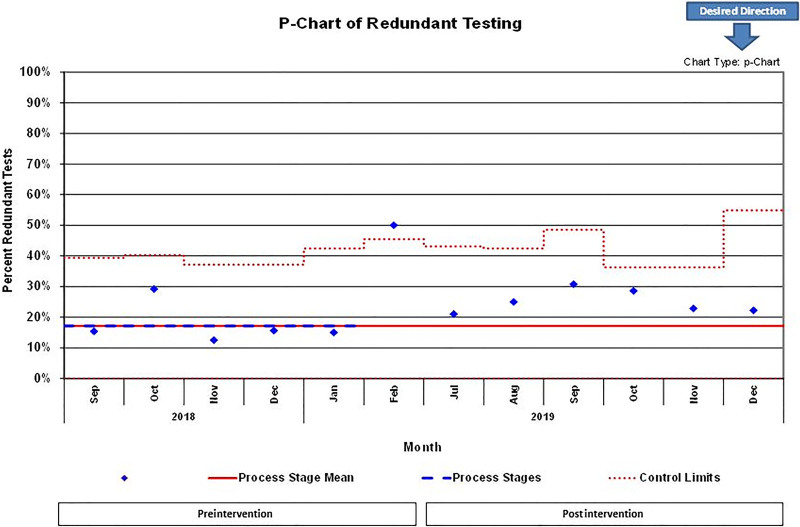
Control P-chart with monthly redundant laboratory and imaging testing rates over time in preintervention and postintervention periods.

In the postintervention period, we screened 222 patients, and 131 met inclusion criteria. Forty-nine patients (37%) had repeated studies, of which 33 (25%) had redundant studies (Fig. 2). A comparison of all patients who had labs repeated revealed no statistical differences concerning demographics or diagnoses (Table 3). The P-chart of the data (Fig. 3) displaying the overall percentage of transferred patients with redundant testing revealed no statistical difference, albeit the postintervention period did show a drift toward greater redundant testing. As in any process, in both the preintervention and postintervention period, common cause variation did lend to some degree of natural variation, yet most data points were within control limits. However, the data point that includes February 2019 reveals an out-of-control point and likely indicates special cause variation. Therefore, we chose not to include it in calculating the preintervention baseline.

**Table 3.** Characteristics of Preintervention and Postintervention Patients with Any Repeated Studies

Variable	Pre (n = 50)	Post (n = 49)	*P*
Redundant studies (%)	62	66	0.675
Age (y, mean)	8.39	8.50	0.993
Sex (% female)	50	43	0.547
Internal transport team (%)	30	33	0.830
Daytime (%)	58	39	0.071
Weekend (%)	64	55	0.416
Cost ($, mean)	83.5	101.2	0.066
Assigned admission MDC (%)	Respiratory (32) Endocrine (22) Toxicology (10) Cardiovascular (8) Neurologic (8) Gastrointestinal (6) Hematologic (6) Ear, Nose, and Throat (3) Infectious (2)	Neurologic (24) Respiratory (22) Endocrine (18) Cardiovascular (16) Toxicology (10) Hematologic (4) Ear, Nose, and Throat (2) Infectious (2) Gastrointestinal (0)	0.316

## DISCUSSION

This study established the first known baseline rate of clinically unnecessary laboratory testing for interfacility patient transfers to an academic tertiary care PICU. Approximately, one-quarter of pediatric patients admitted to the PICU underwent redundant testing. The current study findings are akin to those identified in a pediatric emergency department analysis; however, that was limited to repeat testing in one unit. Hence, repeat testing occurred only in 1%–2% of patients yet, 14% of CBCs, and 7% of coagulation studies were unnecessarily repeated despite previously normal results.^8^ The adult literature has more similar data, whereby 53% of patients transferred between emergency departments underwent repeated testing. In 95% of these patients, the repeated test was considered clinically inappropriate.^3^ Therefore, compared to adult data, our percentage is relatively low.^3–5^

Although the rate of redundant testing was relatively low, the mean cost associated with initial admission studies in patients with redundant studies was significantly higher than those with clinically indicated studies. The difference may be diagnosis-related. We know that patients who present with respiratory illnesses such as bronchiolitis, for example, should not undergo routine laboratory evaluations according to national stewardship guidelines.^9^ On the other hand, those patients who present with an endocrinopathy such as diabetic ketoacidosis are more likely to have a clinically indicated reason for repeated studies. Thus, the admitting diagnosis category may be a key driver to wasteful testing where ambiguous diagnoses such as “Respiratory Disorders,” more prevalent in the redundant group, led to more redundant testing. In contrast, diagnoses such as “Endocrine Disorders,” more prevalent in the clinically indicated group, yielded clinical rationale for repeated evaluations.

Interestingly, this group of patients with redundant studies was significantly younger than those with indicated studies (6.87 versus 10.98 years, *P* = 0.023). One could hypothesize that this may be diagnosis-related as well, knowing that again bronchiolitis, as an example, is mainly a disease of younger children. Additionally, when looking more closely at redundant studies (Table 2), we see that a CBC was the most common study to be repeated when not indicated. Although difficult to ascertain, perhaps when ordering an indicated study such as a basic metabolic panel, providers add a CBC without a clear cause, unaware of the increasing cost or potential blood volume.

The reduction of redundant laboratory testing is essential to improving the quality of care of pediatric patients. In addition to the increased cost of care, there may be an association between unnecessary testing and nonmonetary costs, including radiation exposure, acquired anemia from phlebotomy,^10^ pain, patient or parental anxiety, and time required by nursing and ancillary services. Given the high costs associated with critical care medicine overall, it is even more imperative that clinicians adopt a clinically appropriate “less is more” approach to care. National initiatives such as the Choosing Wisely Campaign promulgate guidance targeting wasteful laboratory testing in the ICU setting. However, there is no specific guidance regarding redundant testing on admission.^11^ Although education and visual reminders alone in this study were ineffective, establishing a baseline rate of redundant testing for interfacility transfers admitted to the PICU can be used as a benchmark for future quality improvement initiatives.

Although many studies show that educational endeavors are the least likely to promote sustained change,^2^ there are examples of some that present education as a means to change behavior. For instance, Piper et al^12^ showed that educational methods could reduce opioid prescribing after pediatric surgery. The relatively low initial rate of redundant testing compared to adults and lack of knowledge around this area made an educational initiative an attractive low-fidelity strategy to test in our setting. The lack of change proves that future endeavors must focus on alternative methods.

There are several limitations to this study. First, this was a single-center study in an urban, academic, quaternary PICU, potentially limiting generalizability. As an academic medical center, the testing rates may be artificially higher than those found in a community hospital. Second, we derived the clinical indications for repeat testing by expert consensus opinion of a single cohort of pediatric intensivists. Another group of experts may have developed different criteria for clinically indicated tests. When examining the intervention in our study, there are some important points to consider. First, process instability exists in our preintervention data as shown by the outlier data point February 2019 in the P-chart (Fig. 3), indicating special cause variation. One reason for this type of variation may have developed from the inherent increase in census during the winter months when providers may have lacked time to pay attention to the timing of outside hospital laboratory work. Furthermore, the trend toward increased redundant testing in the postintervention period, albeit insignificant, may have been due to a Hawthorne effect of research team members collecting data in the unit. We suspect that providers’ inclination to be more conservative with the repetition of labs on transferred patients may have been high during the preintervention period yet waned overtime during the postintervention period despite the intervention.

### Future Directions

Several points deserve further exploration from the outcomes of this study. One of these is the statistically significant age difference in the patients with redundant studies in the preintervention period. Although it may be diagnosis-related, this would need to be better established and perhaps serve as a target population of future projects. Another variable that could be targeted in future endeavors is specific laboratory tests. We found that the most frequent redundant laboratory drawn was a CBC; subsequent initiatives could focus efforts specifically on discouraging the repetition of unnecessary CBCs.

Alternatively, future initiatives could focus on provider inclination for repeat testing. Although these steps were not completed in this study, focused interviews with clinicians shortly after identifying redundant testing could help determine the issue’s root cause. Such discussions and custom changes would likely create more inclination to improve individual practices.

Alternative interventions for changing ordering behaviors are important, and there have been several studies evaluating the effectiveness of different methods. Audit and feedback on ordering practices and restrictive ordering guardrails show meaningful and sustained reduction in laboratory testing in several adult studies.^2^ Meanwhile, in a study by Knighton et al,^13^ an attempt was made to define the rate of repeat testing in a pediatric population to identify risk factors, specifically, the use of multiple health systems. They discovered that patients with repeated testing had a higher probability of using multiple health systems (20.8%) versus those using 1 healthcare system (13.8%).^13^ The impact due to lack of electronic health record interoperability between two institutions providing coordinated care to patients was further revealed in a study by Stewart et al.^14^ The study included pediatric and adult patients with findings suggesting interoperable EMR’s could potentially lead to a reduction in duplicative testing.^14^ These findings suggest that forcing functions and increased access to prior data may be critical elements to reducing wasteful testing.

## CONCLUSIONS

In this single-center study, the rate of redundant laboratory and imaging evaluations on interfacility transfer patients admitted to the PICU was 21%–25%. Patients with redundant testing had significantly higher initial laboratory costs and were younger than those who underwent clinically indicated repeat testing. A low-fidelity educational initiative and visual reminders alone did not reduce the frequency of wasteful testing.

## DISCLOSURE

The authors have no financial interest to declare in relation to the content of this article.
